# Treatment of Sprains

**Published:** 1847-04

**Authors:** 


7. *Treatment of Sprains.*—The means which Dr. Poullain and the above authorities recommend, in lieu of leeching and cataplasms, is the immediate and continual application of cold by immersing the part in water. The cure is not only prompt but complete, inasmuch as there is no remaining engorgement to lay the foundation of future mischief, and the patient is enabled to employ the joint as actively as heretofore. This would be a great point gained even if the time consumed in the treatment were as great in the one plan as in the other, which it is not. Many cases of its success are related in the paper, and although, of course, in the great majority of instances, the ancle is the joint affected, sprains of other joints may be treated in just the same manner, except that in those, such as the knee, in which immersion may be difficult the application of wet compresses or irrigation may be substituted. The treatment, indeed, is not novel, for it was recommended by Boyer, and more recently by M. Begin.

"Of 90 patients whom I have treated by the aid of cold and resolvents, 23 were cured in 6 days, 10 in 8 days, 22 in 11 days, 28 in from 11 to 15 days, 4 in from 20 to 25 days, and three at the end of a month. None of these persons have continued lame. Seven felt the effects of their accident for several months, without, however, being prevented from attending to their duties, and becoming quite cured. * * * * * If this mode of treatment has incurred blame at the hands of some surgeons, it is because it has not been sufficiently, promptly and freely employed, and it is therefore necessary to lay down some rules upon this point.

"The immersion should be resorted to as soon after the accident as possible. Recourse may be had to it also three, four, five, six, or even twelve hours after, but then its sedative effect is less prompt and the cure more tedious. The foot should remain at least *two hours* in the bath, and oftentimes much longer. It may sometimes be left in for entire days; and as a general rule the part should not be removed until it becomes completely cooled, the water being renewed as often as it becomes warm. This prolongation is easily obtained, for, after the first hour or so (during which the pain is sometimes almost insupportable), the immersion becomes bearable and the patient himself very desirable for its continuance. Iced water does not possess any superior efficacy to that of a temperature of 37° or 39°, provided this be equally maintained. As soon as the limb is removed from the bath it must be surrounded by a roller previously moistened with Goulard water or camphorated spirits, some of which must afterwards be applied to it from time to time. So effectually are congestion and swelling in this way diminished, that the bandage usually becomes loose within the twenty-four hours. It must be re-applied until all swelling and pain have disappeared, which is generally the case in from three to six days. The patient may now be allowed to walk, continuing, however, the use of a bandage for ten or twelve days.

"If fourteen, or even six or twelve hours, after the application of the wet bandage, pain still continues, or throbbing is felt by the patient, it must be taken off, and the limb again immersed in the water for a longer period than at first, even for a whole day, if requisite. The second immersion is sometimes unsuccessful, but fortunately it is very rarely required, as the first almost always suffices.

"If the sprain is several days old, the limb swollen and painful, while nothing has been done for it, a free local bleeding is a necessary preliminary, after which the bandage and cold lotions, or perhaps immersion itself, should at once be resorted to. These means are, however, now of far less service than when employed soon after the occurrence of the accident."

When the sprain has been badly treated, the joint may become the seat of a chronic enlargement, which is dissipated with difficulty, and only after the persevering use of compression. MM. Begin and Velpeau, indeed, employ this in the earliest stage of sprain as a powerful means of preventing inflammatory swelling. Dr. Poullain employs to this end a starched many-tailed bandage. Whatever means are used the case is tedious and may also require the aid of stimulating liniments, or of the douche as employed at the mineral springs and even this does not dissipate the enlargement.—*Rev. of Poullain, in the Brit. and F. Rev.*, in *Ibid.*

